# Translation initiation consistency between *in vivo* and *in vitro* bacterial protein expression systems

**DOI:** 10.3389/fbioe.2023.1201580

**Published:** 2023-05-25

**Authors:** Jiaojiao Li, Peixian Li, Qian Liu, Jinjin Li, Hao Qi

**Affiliations:** ^1^ School of Chemical Engineering and Technology, Tianjin University, Tianjin, China; ^2^ Frontier Science Center for Synthetic Biology (Ministry of Education), Tianjin University, Tianjin, China; ^3^ Zhejiang Shaoxing Research Institute of Tianjin University, Shaoxing, China

**Keywords:** cell-free system, gene expression regulation, protein engineering, synthetic biology, *E. coli*

## Abstract

Strict on-demand control of protein synthesis is a crucial aspect of synthetic biology. The 5′-terminal untranslated region (5′-UTR) is an essential bacterial genetic element that can be designed for the regulation of translation initiation. However, there is insufficient systematical data on the consistency of 5′-UTR function among various bacterial cells and *in vitro* protein synthesis systems, which is crucial for the standardization and modularization of genetic elements in synthetic biology. Here, more than 400 expression cassettes comprising the GFP gene under the regulation of various 5′-UTRs were systematically characterized to evaluate the protein translation consistency in the two popular *Escherichia coli* strains of JM109 and BL21, as well as an *in vitro* protein expression system based on cell lysate. In contrast to the very strong correlation between the two cellular systems, the consistency between *in vivo* and *in vitro* protein translation was lost, whereby both *in vivo* and *in vitro* translation evidently deviated from the estimation of the standard statistical thermodynamic model. Finally, we found that the absence of nucleotide C and complex secondary structure in the 5′-UTR significantly improve the efficiency of protein translation, both *in vitro* and *in vivo*.

## 1 Introduction

Tunable genetic elements are crucial for various applications in metabolic engineering and synthetic biology (Chappell et al., 2013). Protein abundance in bacterial cells is determined by factors such as regulatory elements in the RNA, ribosome density (Arava et al., 2005), and selection of the start codon (Basu et al., 2022). Studies have demonstrated that the 5′-untranslated regions (5′-UTRs) (Ding et al., 2018) comprising the core ribosome-binding site (RBS) is a pivotal element controlling the translation initiation efficiency and protein expression level (Salis et al., 2009; Shi et al., 2018) (Seo et al., 2013). Recent studies found that designed 5′-UTRs can control protein translation at a wide range of levels in various expression systems, including yeast (Cuperus et al., 2017), *E. coli* (Duan et al., 2022), and human cells (Sample et al., 2019). Interestingly, these studies indicated that UTRs with a biased nucleotide composition are more conducive to high translation efficiency. In particular, A and U in the UTR sequences significantly contribute to the translation efficiency.

*E. coli* cell is the most common model system for the efficient expression of heterologous proteins because of its well-studied genetic background, fast growth rate, simple genetic manipulation, and high protein expression capacity (Yoon et al., 2012; Marisch et al., 2013; Han et al., 2020). The suitability of *E. coli* for high cell-density culture enables the cheap production of recombinant proteins (Phue et al., 2005; Son et al., 2011; Englaender et al., 2017), while the well-studied metabolic network makes *E. coli* a popular platform for natural product synthesis (Pontrelli et al., 2018). The K-12 and B strains of *E. coli* with their variants are among the most widely used host cells in biological research and industrial fermentation (Marisch et al., 2013), whereby *E. coli* K-12 strains are more widely used in genetic manipulation and biochemical research (Kuhnert et al., 1995; Posfai et al., 2006). According to Northern blot analysis, the mRNA expression level of the *E. coli* B strain is higher than in the K-12 strain (Shiloach et al., 2000; Phue and Shiloach, 2004). Furthermore, *E. coli* B strains, which lack genes for flagella production and express the motility-related proteins at a relatively low level, can achieve a higher biomass yield and faster growth than *E. coli* K-12 strains (Yu et al., 2002; Shiloach et al., 2010). Previous studies have investigated the differences between *E. coli* B and K-12 strains by transcriptomic and proteomic approaches (Marisch et al., 2013), as well as metabolomic analysis (Shiloach et al., 2010).

*In vitro* protein synthesis, CFPS provides high flexibility and controllability. Cell extract-based *in-vitro* systems are used as a platform for rapid characterization of regulatory elements, circumventing the inherently time-consuming and low-throughput manipulation of living cells, in various applications of proteins engineering and synthetic biology (Hodgman and Jewett, 2012; Sun et al., 2014; Lu, 2017). In contrast to the complexity and limitations of living cells, the functional performance of genetic elements can be rapidly validated and screened from libraries using *in vitro* platforms, such as PURE (Shimizu et al., 2001) and lysate-based systems (Kightlinger et al., 2018). Extract-based systems have been utilized to rapidly evaluate different RBSs, showing the inconsistency between the theoretical prediction and actual protein synthesis levels (Wang et al., 2018). Interestingly, recent studies showed that there was also a deviation between the *in vivo* and *in vitro* protein synthesis levels using the same elements (Zhang et al., 2021). Therefore, the systematic characterization of the regulation of protein expression in various strains and even *in vitro* systems is necessary for improving standardization and modularization in synthetic biology.

In this study, we systematically quantified the expression levels of 416 superfolder GFP (sfGFP) expression cassettes with various 5′-UTR sequences in two *E. coli* strains, BL21 Star (DE3) and JM109 (DE3), as well as an *in vitro* lysate-based system to evaluate the consistency of UTR function. In contrast to the strong correlation between these two cellular systems, the consistency between *in vivo* and *in vitro* protein translation is lost, whereby both *in vivo* and *in vitro* translation levels evidently deviate from the estimation of the standard statistical thermodynamic model. Specifically, we also quantified the function of 5′-UTRs comprising only three types of nucleotides. Finally, we found that the absence of C nucleotides and a complex secondary structure in the 5′-UTR significantly improve protein translation, both *in vivo* and *in vitro*. Therefore, we believe that designing genetic elements with high consistency across various strains and even *in vitro* systems is necessary for effective standardization and modularization in synthetic biology.

## 2 Materials and methods

### 2.1 Bacterial strains, plasmids, and growth conditions

The *E. coli* strain JM109 (DE3) [*end*A1, *gln*V44, *the-1*, *rel*A1, *gyr*A96, *rec*A1, *mcr*B+, Δ (lac-proAB), e14- (F’ *tra*D36 *pro*AB, *lac*IqZΔM15), *hsd*R17 (rk^−^, mk+), + λ (DE3)] and the *E. coli* strain BL21 Star (DE3) [F^−^ *omp*T *hsd*S_B_ (r_B_^−^, m_B_^−^) *galdcmrne*131 (DE3)] were used as host organisms and cultivated in Luria−Bertani (LB) medium supplemented with the working concentration of 100 μg/mL ampicillin at 37°C for the 5′-UTRs activity validation. Primers for amplification were synthesized by Azenta. The pUC19 plasmid and pUC19-wt-sfGFP plasmid were constructed by our laboratory, and the recombinant plasmids with 5′-UTR sequences were verified by Sanger sequencing. All primers are listed in Supplementary Table S1.

### 2.2 Construction of randomized libraries

We generated a library of plasmids with 5′-UTRs containing a 25 nucleotides long randomized region directly upstream of the start codon. Firstly, the upstream primer 25N-F and downstream primer 25-R were synthesized, annealed and amplified using the Primer STAR Max premix (TaKaRa). The PCR was performed with one cycle of 98°C (10 s), 55°C (5 s), and 72°C (1 h) followed by cooling to 10°C. Secondly, the linearized vector was obtained from the original plasmid pUC19-nonRBS-sfGFP constructed in our laboratory by PCR amplification using the V19-F and V19-R. Then, the amplified PCR products were purified using a Tian quick PCR Purification Kit (Qiangen) or Tian quick Gel Extraction Kit (Qiangen). Finally, the recombinant plasmids were assembled using the ClonExpress II One Step Cloning Kit (Vazyme Biotech Co., Ltd.) and transferred into the competent *E. coli* JM109 (DE3) cells. Libraries were cultivated overnight on the LB plate with ampicillin for until single colonies appeared. The cultured plates were placed in a refrigerator at 4°C for the subsequent activity measurements.

### 2.3 Characterization of 5′-UTRs in live *E. coli* cells

The growth and fluorescence measurements were performed in 96-well high-throughput format. Single colonies were picked from the plate and cultivated overnight in LB medium with 100 μg/mL ampicillin at 37°C, 14,000 rpm for 12 h in the deep well maximizer (TAITEC, MBR-022UP). Then, 160 variants with different 5′-UTR regions were selected by Sanger sequencing to remove the repeat sequences. There were three positions (wells) in each 96-deep-well plate for the controls, including the negative control in the H10 position, the positive control in the H11 position and the blank control in the H12 position. The cells were cultured overnight, and seeded at a ratio of 1:100 into the 96 deep-well plate (VIOX scientific) containing 300 μL liquid LB medium with 100 μg/mL ampicillin and 0.5 mM IPTG per well, followed by culture at 37°C, 14,000 rpm for 5 h. After that, 80 μL of culture was taken out with an electronic pipette (Eppendorf Xplorer^®^) and placed it into a 96-well plate (Corning#3762) which contained 120 μL of LB medium in each well. We measured the optical density (OD_600_) and fluorescence value of each culture (485/535 nm) using a microplate reader (Tecan Spark multimode microplate reader). In the process of activity measurement, we measured each sample three times and calculated its average value. For the correlation analysis of 5′-UTRs in the living cell system, the absolute fluorescence for each 5′-UTR sequence was calculated and normalized to the OD_600_. For the correlation analysis of 5′-UTRs between *in vivo* and *in vitro* systems, we respectively calculated their relative fluorescence according to the formula (Salis, 2011):

$${FLU}_{sample}=\frac{{FLU}_{sample}-{FLU}_{media}}{{OD}_{sample}-{OD}_{media}}-\frac{{FLU}_{puc19}-{FLU}_{media}}{{OD}_{puc19}-{OD}_{media}}$$ (1)

where the FLU_sample_, FLU_media,_ and FLU_pUC19_ respectively represent the sfGFP fluorescence of the sample, the blank control, and the negative control, while the OD_sample_, OD_media,_ and OD_pUC19_ respectively represent the OD_600_ of the sample, the blank control, and the negative control.

The relative fluorescence value per cell concentration of wild-type cells was calculated according to the formula:

$${FLU}_{wt}=\frac{{FLU}_{wt}-{FLU}_{media}}{{OD}_{wt}-{OD}_{media}}-\frac{{FLU}_{puc19}-{FLU}_{media}}{{OD}_{puc19}-{OD}_{media}}$$ (2)

where FLU_wt_ represents the sfGFP fluorescence of the positive control, and OD_wt_ represents the OD_600_ of the positive control.

The relative activity of each sample was standardized to the FPLC_wt_ for the comparisons of different 5′-UTRs. Here, we defined the relative intensity of the 5′-UTR region as the percentage of the relative fluorescence value per cell concentration of the sample and the relative fluorescence value per cell concentration of wild-type cells, according to the formula:

$$P\%=\frac{{FPLC}_{sample}}{{FPLC}_{wt}}\times 100\%$$ (3)

According to the above calculation method, we measured and analyzed the real relative activity of 93 sequences with different 5′-UTRs in the *E. coli* JM109 (DE3) and BL21 Star (DE3) strains.

### 2.4 Batch extraction of plasmids and bacterial transformation

The direct boiling method for plasmid DNA extraction was adopted as described before (Peng et al., 2013), with minor modifications as follows. The cryostock containing the strain library was re-cultured at 37°C and 14,000 rpm for 12 h in the 96 deep-well plate. Then, 70 μL samples from the 96 deep-well plate were added into eight consecutive rows of PCR tubes with corresponding position labels. The *E. coli* cells were collected by centrifugation at 6,000 rpm for 10 min with a high-speed refrigerated microcentrifuge (MDX-310, LTD.), and added to 100 μL of ddH_2_O, followed by vigorous vortexing to homogenize the suspension. Then, the cell suspensions were incubated at 95°C for 10 min and subsequently centrifuged at 14,000 rpm at room temperature for 15 min. Finally, 50 μL of the supernatant were transferred to a clean 96-well PCR plate and stored at −40°C.

The competent *E. coli* BL21 Star (DE3) cells were prepared by the calcium chloride method (Seidman et al., 2001), and packed into 96-well plates at 100 μL/well for transformation. The amount of the obtained plasmid plays an important role in the successful transfer process, considering the purity and the transformation efficiency. Hence, we carried out a series of optimization experiments and determined the optimal amount (Supplementary Figure S1A). Then, the competent cells were incubated on ice for 30 min. Heat-shocked for 45 s at 42°C in an electric constant temperature water bath (DK-98-ⅡA Tianjin TEST Instrument Co., Ltd.), and immediately placed in an ice bath for 2 min. Subsequently, 700 μL SOB medium was added and the cells were recovered at 37°C and 14,000 rpm for 3 h. Finally, 30 μL of the cells were pipetted into 300 μL of liquid LB medium containing 100 mg/mL ampicillin and cultured overnight at 37°C and 14,000 rpm.

### 2.5 Characterization of 5′-UTRs in a cell-free system

The S30 cell extract was prepared as described in our previous study (Wu et al., 2022), but we did not carry out the run-off reaction for high CFPS yields. Hence, the subpackage extract was directly frozen in liquid nitrogen and stored in the refrigerator at −80°C.

To prepare the DNA template for CFPS reaction, a standard PCR reaction for each sample in a 96-well plate was performed in a 25 μL system comprising 1 × Easy Taq Buffer (TransGen, ET101), 0.2 mM dNTPs, 2.5 U Easy Taq polymerase (TransGen Biotech), 1 μL extracted DNA template, 0.2 mM forward primer F2 and 0.2 mM reverse primer R2. Thermocycling conditions were as follows: 10 min at 94°C; 30 cycles of 30 s at 94°C, 30 s at 55°C, and 60 s at 72°C, followed by a 5 min extension at 72°C. The PCR products were analyzed by electrophoresis on a 2% agarose gel to confirm the size of the band and subsequently stored in the refrigerator at 4°C.

For the characterization of 5′-UTR *in vitro*, a standard CFPS reaction in the 20 µL reaction system was used containing 2 μL of PCR products and other reaction components same as described in a previous report (Wu et al., 2022). The addition of 2 μL of the PCR products was optimal for achieving the highest yield in this CFPS reaction (Supplementary Figure S4B). All reactions were correspondingly pipetted into a 384-well plate (Corning #3762) and transferred to a microplate reader with real-time fluorescence monitoring (excitation at 485 nm and emission at 535 nm) for up to 3 h in 5-min intervals at 30°C, shaking linearly for 5 s before each measurement. The reported data are the averages of three independent measurements. The background fluorescence intensity from a cell-free reaction with the empty plasmid (pUC19) was subtracted from each sample fluorescence measurement, and the resulting intensity values were normalized to the positive control plasmid (pUC19-wt-GFP) to calculate the relative expression strength.

### 2.6 Analysis of 5′-UTRs sequences

The sequence logo for the 5′-UTR library was generated using the online analysis tool WebLogo (http://weblogo.berkeley.edu/). The translation initiation strength of each 5′-UTR was predicted using the RBS Calculator (Salis et al., 2009). For the secondary structure analysis of 5′-UTRs, minimal folding energies (MFE) were calculated for the region encompassing the entire 25 nt 5′-UTR and 27 nt downstream of the sfGFP coding region using NUPACK 4.0. To estimate the recognition of the 5′-UTR region by the 16S rRNA 3′-terminal region, we also calculated the free hybridization energy of 5′-UTR fragments with the anti-SD sequence (5′-ACCUCCUUA-3′) using NUPACK 4.0 (Zadeh et al., 2011).

### 2.7 Statistical analysis

All r values are Pearson correlation coefficients of the strength of the linear relationship between the two sets of data. All scatter plots and histograms were generated using the Origin software package. Quantitative data are presented as means ± standard deviations (SD) from three experiments.

## 3 Results and discussion

### 3.1 Characterization of 5′-UTRs in different bacterial strains

**FIGURE 1:**
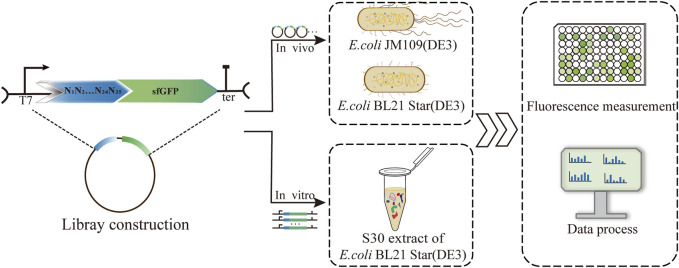
Establishment and characterization of a 5′-UTR library based on three protein expression platforms, including the *E. coli* K-12 and B strains and a cell-free system. Correlations of the translation initiation strengths of the 5′-UTRs in the different systems were analyzed via sfGFP fluorescence measurements.

For systematic quantification of the consistency of translation efficiency of 5′-UTRs, we established different protein expression platforms, including the living cell system and cell-free system. The 5′-UTRs library was constructed and measured correspondingly the protein initiation expression levels. Correlations of the translation initiation strengths of the 5′-UTRs in the different systems were analyzed via sfGFP fluorescence measurements (Figure 1). For the validation of 5′-UTRs consistency, the *E. coli* K-12 and B strains were selected as the protein expression platforms *in vivo*. To assess the translation efficiency in *E. coli* K-12 and *E. coli* B, we measured the fluorescence and analyzed their correlation. According to the ribosome profiling studies (Kim et al., 2012), the average length of 5′-UTRs in *E. coli* cells is 25–30 nt. Firstly, we constructed a library of 5′-UTRs, with unbiased 25 N random nucleotides, where N stands for any of A/C/G/T. The 5′-UTRs library was cloned into a plasmid encoding the sfGFP reporter gene to determine the translation initiation strength by measuring the fluorescence. Therefore, the unbiased 5′-UTR library was introduced into *E. coli* JM109 (DE3) cells cultured on solid LB medium with ampicillin. Then, 160 single colonies with unique 5′-UTRs sequences were randomly picked from the plates for the analysis of sfGFP translation efficiency by measuring the corresponding fluorescence.

**FIGURE 2:**
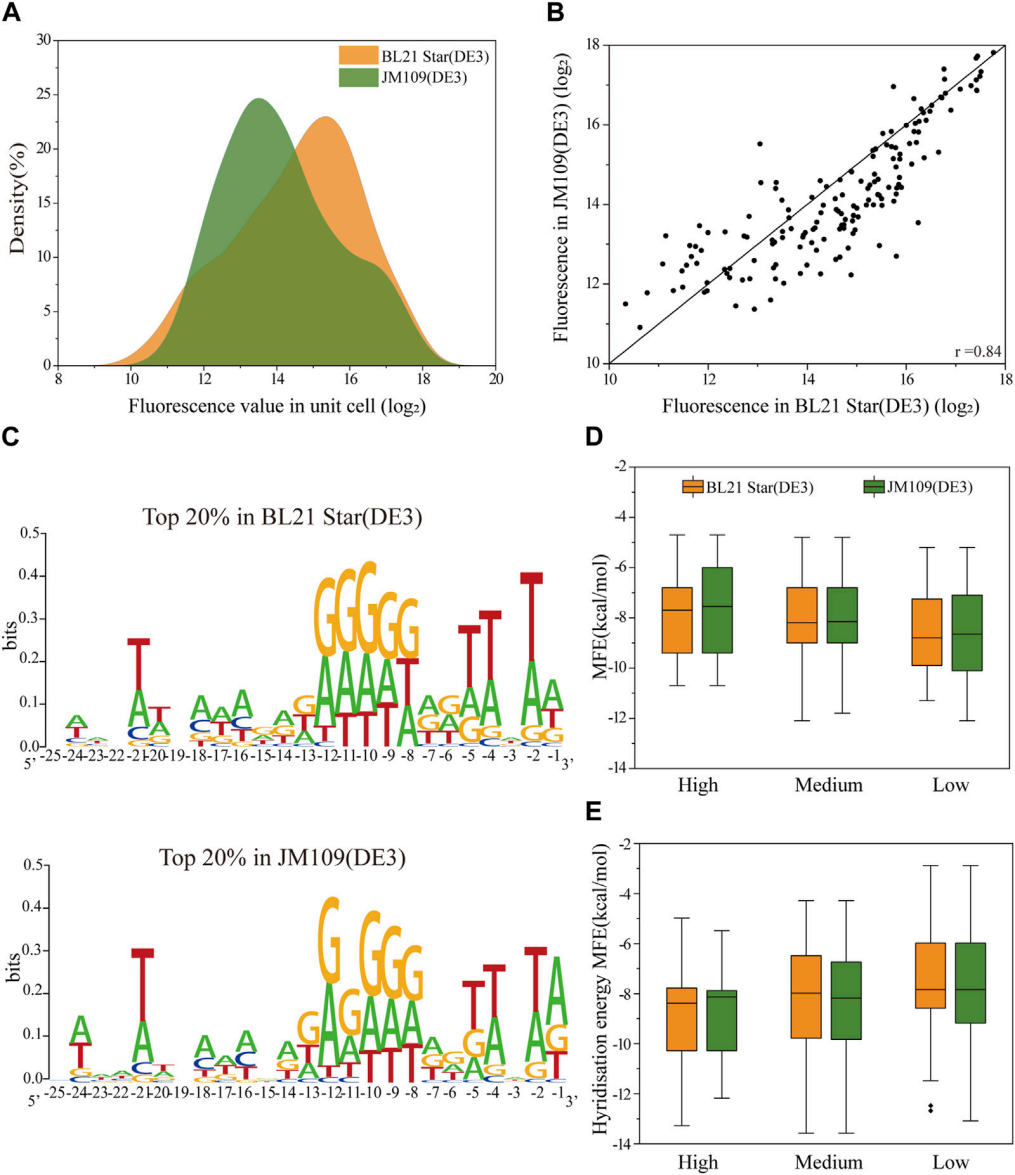
The correlation of the translation initiation strengths of the unbiased 5′-UTR library in *E. coli* JM109 (DE3) and BL21 (DE3) *in vivo*. **(A)** The distribution of absolute fluorescence values of the unbiased 5′-UTR library containing 160 unique sequences in both strains. **(B)** The correlation of absolute fluorescence of 160 5′-UTR variants between the two strains (Pearson’s r = 0.84). **(C)** Sequence logos were calculated for the Top 20% tested sequences in each strain. **(D)** Distributions of the minimal free energy (MFE) of the secondary structure folding for 5′-UTRs with different fluorescence levels between the two strains. **(E)** Distributions of the minimal hybridization energy of the 5′-UTR fragments with the anti-SD sequence ACCUCCUUA at the 3′end of the 16S rRNA between the two strains. We defined absolute fluorescence levels above 16.2 as high activity, those absolute fluorescence levels between 13.2 and 16.2 as medium activity, and those below 13.2 as low activity.

To analyze the correlation of the translation initiation strength of the 5′-UTRs in different strains, the plasmids containing the 160 5′-UTRs variants were extracted and transferred into *E. coli* BL21 Star (DE3) cells in batch (see Materials and methods 2.4 for a specific description). The optimal volume of the plasmid DNA of the transformation was quantified to be 8 μL (Supplementary Figure S1A). For each cell sample, the average fluorescence normalized by cell density (measured absorbance at 600 nm) was quantified for the sfGFP translation efficiency. We plotted the distribution of absolute fluorescence of the 160 unbiased 5′-UTRs sequences measured in both *E. coli* JM109 (DE3) and *E. coli* BL21 Star (DE3) cells (Figure 2A). The absolute fluorescence of the two strains overall spanned a similar range. In addition, the number of BL21 Star (DE3) cells with high activity was significantly higher than JM109 (DE3) cells. The analysis of the activity distribution for all 160 unbiased 5′-UTRs sequences revealed that *E. coli* B strains, as chassis cells, were more favorable than K-12 for the production of heterologous proteins. This result could be explained by the inherent properties of the *E. coli* B strain, as was previously indicated through the comprehensive analysis of multi-omics data, including the genome, transcriptome, proteome, and phenome data (Yoon et al., 2012). The elements involved in the amino acid biosynthesis pathway and the absence of genes for flagella and proteases could contribute to the beneficial metabolism and physiological state of *E. coli* B.

More importantly, with regard to the 5′-UTRs translation regulation, we found a significant correlation in between the *E. coli* JM109 (DE3) and BL21 Star (DE3) cells (Pearson’s r = 0.84; Figure 2B). It implied that the regulatory properties of the 5′-UTRs in *E. coli* K-12 or B strains are universal. Considering the measured absolute fluorescence, we measured the respective growth curves of each strain and the strain containing the benchmark plasmid (Supplementary Figure S1B). From the perspective of the strain’s inherent characteristics, the growth rates were consistent, but showed that strain B grew a little faster than strain K. (Luli and Strohl, 1990). In contrast, the *E. coli* JM109 (DE3) cells transformed with the positive plasmid grown faster than the corresponding *E. coli* BL21 Star (DE3) cells. In general, the *E. coli* B cells were primarily intended for the synthesis of exogenous recombinant proteins, which is why it has a lower growth rate and tends to use more energy for protein synthesis (Bentley et al., 1990; Carneiro et al., 2013). The high consistency of the expression levels in the two strains was attributed due to a number of reasons. Firstly, there is more than 92% similarity among aligned regions of the genome between *E. coli* JM109 (DE3) and BL21 Star (DE3) (Jeong et al., 2009). Moreover, similar regulation in *E. coli* K-12 and B strains was previously demonstrated through comprehensive transcriptomic and proteomic data analysis (Marisch et al., 2013). Overall, the translation initiation strength of 5′-UTRs in both industrial hosts showed high similarity, which provides a valuable understanding for designing microbial cell factories in the future.

Previous analyses have demonstrated that mRNA secondary structures as well as the context and position of 5′-UTRs influence the efficiency of protein translation (Evfratov et al., 2017; Duan et al., 2022). To elucidate the high correlation between the two strains, we implemented and analyzed several elements, encompassing context-dependence and secondary structures. We calculated sequence logo for total of 160 5′-UTRs sequences after sequencing the corresponding regions from all the strains (Figure 2C). It is generally understood that adenosine and guanine showed high conversation levels at positions −7 to −12, approximately close to 0.3 bits. As expected, the characteristics of the sequence in this conserved region are consistent with SD sequence features and may be an SD-like sequence (Li et al., 2012). In addition, we also respectively analyzed the nucleotide frequencies at positions −25 to −1 relative to the start codon for the top 20% sequences in the total random library for differences between *E. coli* JM109 (DE3) and *E. coli* BL21 Star (DE3) (Figure 2C). This visible result demonstrated that compositional bias toward A and T at positions −2, −4, and −5 of the 5′-UTR were the consistent characteristic in both strains, which demonstrated that A-U enhancer interacted with ribosomal protein S1 to promote protein expression (Komarova et al., 2005; Duan et al., 2022). As expected, 5′-UTR variants with higher protein expression contained sequences more similar to the SD and SD-like sequences. Overall, the 5′-UTR sequence contents dependence in both strains was generally adapted for the precise tuning of protein expression.

Next, we assessed whether there was a consistent effect of secondary structure on the translation efficiency for protein expression in the K-12 and B strains. To calculate the predicted minimum free energy (MFE) of the 5′-UTRs, we used the NUPACK algorithm to fold the 5′-UTRs sequence along with the first 27 nt of the sfGFP coding region. We classified the protein expression levels into three sets of high, median, and low expression, and then plotted the relationship between MFE and the protein expression level. Binning the 5′-UTRs by their MFE, we found that the higher MFE fraction corresponded to increased protein expression in both K-12 and B strains, implying no difference in the effect of MFE on translation in the two different strains (Figure 2D). Hence, a stable secondary structure of mRNA might mostly downregulate the protein expression, which was not affected by the properties of the cells themselves. In addition, the binding interaction between the SD region of 5′-UTR and the 3′ end regions of 16S rRNA could primarily determine the translation initiation process (Shine and Dalgarno, 1974). The free hybridization energy of the 5′-UTR fragments with the anti-SD sequence ACCUCCUUA at the 3′ end of the 16S rRNA was calculated for the correlation analysis of protein expression between the *E. coli* JM109 (DE3) and BL21 Star (DE3) strains. Lower hybridization energy was correlated with higher fluorescence in both *E. coli* strains (Figure 2E). These results demonstrated that the interaction of the ribosomes with the 5′-UTRs did not result in any difference in the evaluation of protein expression between *E. coli* K-12 and B. In addition to the local mRNA structure, the N-terminus sequence around the translation start sites in coding protein regions affected the translation initiation efficiency (Kudla et al., 2009; Goodman et al., 2013). Hence, we selected five 5′-UTR sequences with high, medium and low translation efficiency respectively and fused them with another protein, Glutathione-S-transferase (GST), to evaluate the translation initiation consistency in *E. coli* BL21 Star (DE3) strains. In *E. coli* B cells, the expression of GST and sfGFP showed a medium correlation (Pearson’s r = 0.75; Supplementary Figure S2). This result suggested that the regulation consistency of the same 5′-UTR sequence’s translation initiation efficiency for different reporter proteins was correlated, but the correlation consistency was lower than that for different types of cells under the same reporter protein condition. This indicated that considered codon distribution in different reporter protein sequences may play a crucial role in the consistency of 5′-UTR regulation (Verma et al., 2019). In summary, the high correlation of the 5′-UTR characteristics between the K-12 and B strains in multiple data analyses provided a general rule for the assignment of 5′-UTR elements.

### 3.2 The nucleotide content of the 5′-UTR influences translation initiation

**FIGURE 3:**
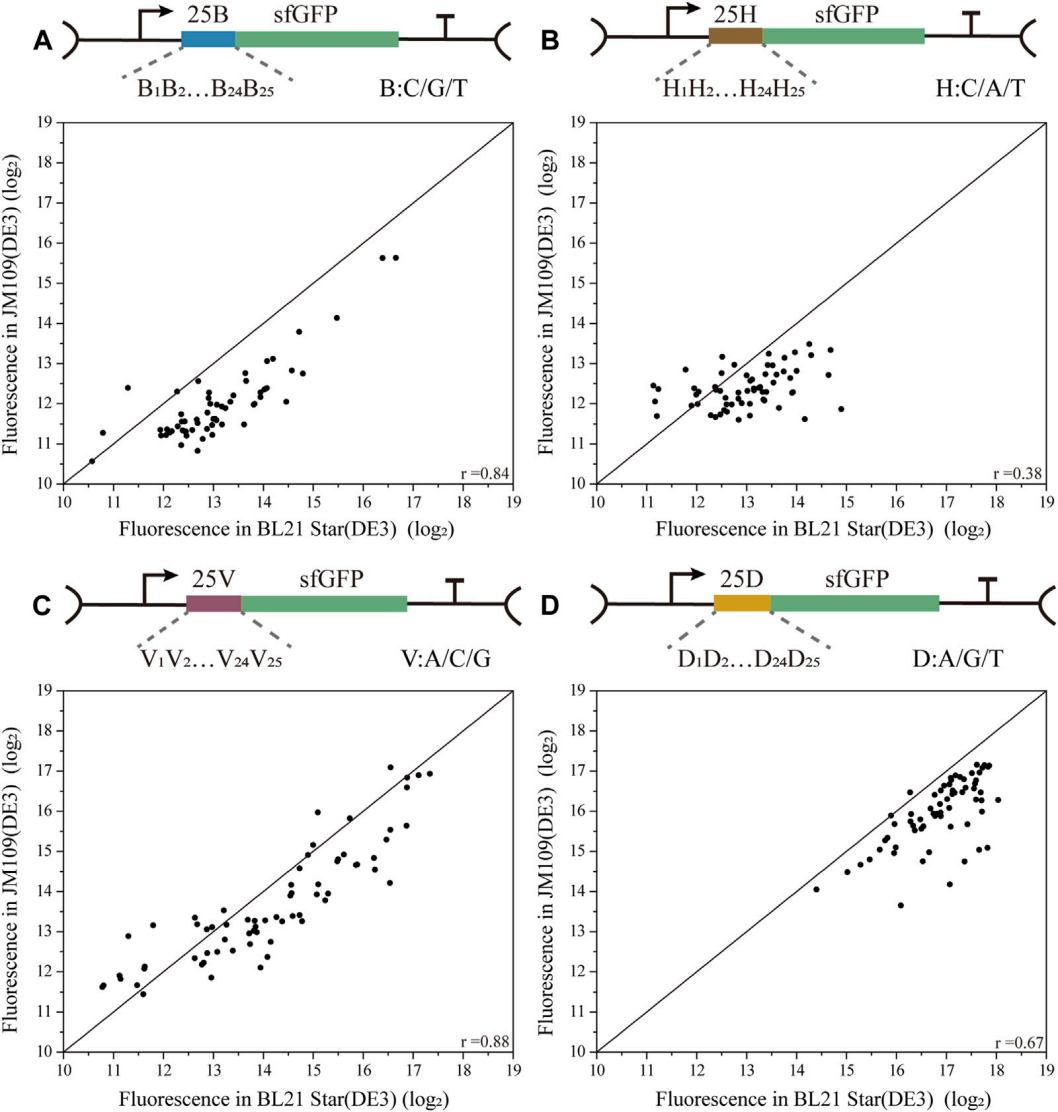
Correlation analysis of four biased 5′-UTR libraries in *E. coli* BL21 Star (DE3) and JM109 (DE3). **(A)** The 25 B library contained 59 unique 5′-UTRs with 25 continuous degenerate bases B, where B stands for C/G/T (Pearson’s r = 0.84). **(B)** The 25H library contained 63 unique 5′-UTRs with 25 continuous degenerate bases H, where H stands for C/A/T (Pearson’s r = 0.38). **(C)** The 25V library contained 68 unique 5′-UTRs with 25 continuous degenerate bases V, where V stands for A/C/G (Pearson’s r = 0.88). **(D)** The 25D library contained 66 unique 5′-UTRs with 25 continuous degenerate bases D, where D stands for A/G/T (Pearson’s r = 0.67). The correlation analysis of these four libraries between BL21 Star (DE3) and JM109 (DE3) is based on the analysis and comparison of absolute fluorescence values.

To decipher the correlations among biased 5′-UTR library characterization in the two *E. coli* strains, four biased 5′-UTR libraries were designed by introducing 25 degenerate bases (B/H/V/D), where C/G/T was designated as B, A/C/T was designated H, A/C/G was designated as V, and A/G/T was designated as D (Figure 3). The four resulting biased 5′-UTRs libraries were first introduced into the JM109 (DE3) cells, and picked colonies were then sequenced and then transferred into the BL21 Star (DE3) strain (see methods) as well. Finally, the absolute fluorescence of the four biased 5′-UTR libraries in both strains was measured for the correlation analysis. Notably, we discovered that there was a high variability in the consistency of the translation initiation strength between the two strains for these four libraries (Figure 3). Especially the 25H library showed a lower association with the protein expression in both *E. coli* strains (Pearson’s r = 0.38 for 25H library), and the corresponding fluorescence levels were relatively low, whereby the JM109 (DE3) strains spanned a narrow range level (Supplementary Figure S3B). Moreover, the weak performance of the 25H library illustrates that guanine dependence of 5′-UTRs can lead to remarkable variations of protein translation in different chassis hosts. There is an urgent need for 5′-UTRs with low expression for the engineering of the metabolic pathways, which can be rapidly selected from a biased library, such as the above-mentioned 25B library. However, the 25B and 25V libraries showed a higher correlation of protein expression in both *E. coli* strains (Pearson’s r = 0.84 for 25B, Pearson’s r = 0.88 for 25V). More importantly, the 25B and 25V libraries showed the interval distribution of measured activity in two chassis strains was uniform, which is close to the normal distribution (Supplementary Figure S3A, S3C). In other words, the 5′-UTR sequences lacking A and T exhibited high versatility between *E. coli* K-12 and B strains for the measurement of translation efficiency. This interesting phenomenon means that A and T context dependence contributes weakly to the correlation between the biased library and host cells. Significantly, the 25D library of 5′-UTRs performed with medium consistency in protein expression levels between K-12 and B strains (Pearson’s r = 0.67 for 25D), which is shown that mainly exhibit high fluorescence levels (Supplementary Figure S3D). In agreement with a previous study (Evfratov et al., 2017), a low proportion of cytidine residues promoted the efficiency of translation. This result reinforces the notion that target sequences with higher translation efficiency might be screened rapidly from a biased library of 5′-UTRs with the deletion of cytidine residues, which reduced the time and cost of optimization to a certain extent. Therefore, we demonstrated that there is an obvious bias in the distribution and consistency of the regulatory strength of the 5′-UTR sequences for translation in different cells.

### 3.3 Comparison of 5′-UTR functions between *in vivo* and *in vitro* protein synthesis

**FIGURE 4:**
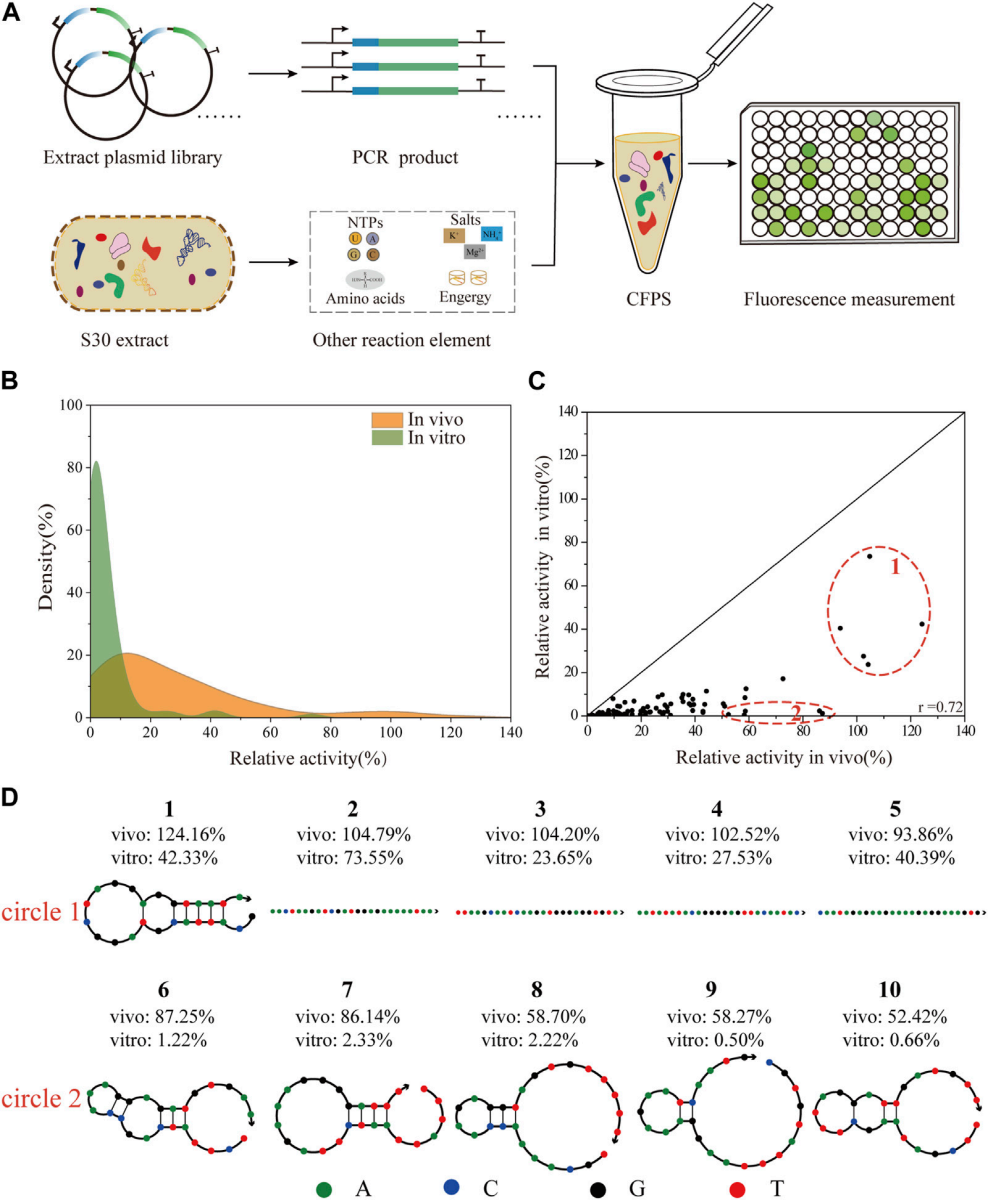
Correlation analysis of the translation initiation strengths of 5′-UTRs between *in vivo* and *in vitro* systems. **(A)** Scheme of the vitro protein expression system. The CFPS system was performed in a single tube containing the S30-based extract, energy sources, nucleotides, amino acids, salts, cofactors, linear DNA, and water/buffer to maintain the reaction. The expression level of sfGFP was determined by direct fluorescence measurement. **(B)** The distribution of the relative translation activities of 93 unique 5′-UTRs between *in vivo* and *in vitro* system. **(C)** The correlation analysis of 93 unique 5′-UTRs sequences in BL21 Star (DE3) *in vivo* and *in vitro* (Pearson’s r = 0.72). **(D)** The secondary structures of five sequences (number 1- number 5) with the highest activity *in vivo* and *in vitro*, and five sequences (number 6—number 10) with high activity *in vivo* but low activity *in vitro* were respectively selected.

To facilitate the engineering of biological elements for cell-free systems, we tested the consistency of 5′-UTRs regulation *in vivo* and *in vitro* (Figure 4A). With the goal of constructing a robust, high-yielding CFPS system, a reported protocol (Wu et al., 2022), was adopted to prepare S30-extracts derived from the cells of *E. coli* JM109 (DE3) and BL21 Star (DE3). To assess the potential ability of protein synthesis, we subsequently carried out the CFPS of the standard sfGFP template in a 20 μL batch reaction for 3 h at 30°C. Representative time courses of sfGFP synthesis using the purified PCR products with online fluorescence measurements were shown in Supplementary Figure S4A. According to the experimental data, the protein synthesis level of the BL21 Star (DE3) extract system was almost 10-fold higher than that of the JM109 (DE3) lysate system. Consistent with previous studies (Kwon and Jewett, 2015), the extract from the *E. coli* K-12 strain showed a lower protein expression level, which could be improved by modifying a few parameters. The S30 extract derived from the BL21 Star (DE3) cells could inherently enable higher protein yields, owing to its genome modification (Jiang et al., 2021). Therefore, we applied the *E. coli* BL21 Star (DE3) extract-based CFPS system to validate the correlation of the whole library of 93 5′-UTR variants between the living cell system and *in vitro* system. After demonstrating high-yield protein expression, we set out to test the feasibility of directly using the PCR amplicon as the CFPS template for high-throughput synthesis. In the gradient addition of the PCR amplicons, we found that 2 µL of the PCR products resulted in close to 49% of the sfGFP synthesis level obtained using the purified PCR product (Supplementary Figure S4B). However, this lower expression level resulted from the instability of the template and salt composition of the PCR mixture (Wang et al., 2018). Emerging strategies for improving the sfGFP synthesis yields, such as increasing the template length (Wu et al., 2007; Hong et al., 2014), or introducing specific chemical modifications (Hong et al., 2014; Sun et al., 2014), and DNA-binding proteins (Yim et al., 2020), did not achieve the desired result. To avoid laborious cloning and purification steps, we decided to directly use the PCR amplicons for measuring the translation initiation strength of the 5′-UTRs.

In theory, as the rate of 5′-UTR translation initiation increases, the protein expression level should increase accordingly. Here, we selected 93 5′-UTR variants from the random library and measured the sfGFP expression in a CFPS platform. To compare the influence of the 5′-UTRs on this different system, the experimental results were respectively normalized to the activity of the positive sequence. As shown (Figure 4B), the distributions of the relative translation activities between *in vivo* and *in vitro* systems were different. Overall, the 93 selected sequences were successfully expressed covering a 30-fold range of translation intensities, and most exhibited lower relative activity using the *in vitro* system. This result was consistent with previous reports that the PCR products were more susceptible to degradation by native exonucleases in the CFPS system (Michel-Reydellet et al., 2005; Seki et al., 2009). Moreover, the relative activity of the 5′-UTR random library showed a medium correlation in tuning the protein expression level between *in vivo* and *in vitro* systems (Pearson’s r = 0.72, Figure 4C). Similarly, previous studies described a clear correlation between the relative strength of an RBS in *E. coli* cells and a CFPS system based on the cell extract (Chappell et al., 2013; Sun et al., 2014). More complex biological engineering, such as genetic circuit design (Sun et al., 2014) and the optimization of biosynthetic pathways (Liu et al., 2020) are also studied in cell-free systems. Additionally, based on high relative activity *in vivo*, the secondary structures of the five 5′-UTRs sequences that showed the highest and lowest activity *in vitro* were respectively identified and analyzed using NUPACK (Figure 4D), an online software for the prediction of nucleic acid structures. Compared to those with higher relative activity *in vitro*, the secondary structures of 5′-UTRs with lower relative activity were more complex (Osterman et al., 2013). As expected, 5′-UTRs with a weaker secondary structure performed the translation more efficiently, which could contribute to the interaction between the mRNA regions and the ribosome during the translation initiation process. This result implied that mRNAs containing 5′-UTR sequences with weak secondary structures tend to be translated more effectively *in vitro*, even though they are potentially more susceptible to degradation by exonucleases.

However, the related research indicated that there is a gap between the vivo prediction and the real relative activity of *in vitro* systems, which were based on different bacterial chassis strains (Wang et al., 2018; Zhang et al., 2021). The mentioned theoretical prediction results for 5′-UTRs were provided by the RBS calculator, which is a powerful platform enabling rational control of protein expression levels (Salis et al., 2009; Salis, 2011). Consistent with previous studies, we also observed a lower correlation between the *in vitro* and *in vivo* prediction results for the *E. coli* BL21 Star (DE3) strain (Pearson’s r = 0.31, Supplementary Figure S5A). As further validation, the correlation of the actual translation initiation strength of 5′-UTRs in *E. coli* BL21 Star (DE3) with their corresponding predictions was low (Pearson’s r = 0.36, Supplementary Figure S5B). This indicates that the influence of the 5′-UTRs on gene expression was not perfectly captured by the RBS calculator. It should be noted that the utilized promoter and plasmid were not suitable for the physical environment in the host cell system. The RBS calculator uses a theoretical thermodynamic model of Gibbs free energies of ribosome binding. While the optimal predicted sequence length before the start codon is 35 nucleotides in the RBS calculator, we only randomized 25 nucleotides. Overall, the observed differences between *in vitro* and *in vivo* systems may be due to disruption of the potential transcription and translation pathways and provides a general strategy for the design of regulatory elements in synthetic biology. In general, these results indicated that although cell-free systems can provide the advantage for rapid prototyping, further optimization of the cell-free systems needs to improve the directly reflective performance *in vivo*. Therefore, the development of an emerging platform for the precise prediction of 5′-UTRs should pay more attention to actual implementation in different expression systems (Na and Lee, 2010; Seo et al., 2013; Pandi et al., 2022). Looking forward, a novel strategy for characterizing biological systems based on the CFPS platform can provide a valuable tool for advancing the development of metabolic engineering and synthetic biology, similar to the comprehensive i^3^-screening pipeline (Kohyama et al., 2023).

## 4 Conclusion

In this study, we assessed the correlation of the translation initiation strength for diverse 5′-UTRs between *in vitro* and *in vivo* systems, which demonstrated differences in the distribution of relative activity and offered explanations of correlation analysis using the Pearson value. For the unbiased 5′-UTR library, a significant correlation of the absolute fluorescence between the *E. coli* K-12 and B strains was observed (Pearson’s r = 0.84). For the biased 5′-UTR library, there were different degrees of correlation between two different types of chassis cells. In addition, an intermediate level of correlation of the relative activity of the 5′-UTR random library was demonstrated between the *in vivo* and *in vitro* systems (Pearson’s r = 0.72). This result implies that there is some degree of deviation from 5′-UTR consistency between the living cell system and the cell-free system. More importantly, our analysis revealed that the lack of nucleotide C and complex secondary structure features in the 5′-UTR are beneficial to enhance the efficiency of protein expression in the different expression systems. Further studies can combine the flexibility and simplicity of cell-free systems to facilitate the manipulation of genetic elements for metabolic engineering and synthetic biology.

## Data Availability

The datasets presented in this study can be found in online repositories. The names of the repository/repositories and accession number (s) can be found in the article/Supplementary Material.
