# Supplementary material for: Translation initiation consistency between in vivo and in vitro bacterial protein expression systems
Source: Front Bioeng Biotechnol. 2023 May 25;11:1201580. doi: 10.3389/fbioe.2023.1201580 (PMC10248181; doi:10.3389/fbioe.2023.1201580)
Supplement: Supplementary file 1 [file DataSheet1.docx]

Supplementary Material

Article Title

First Author*, Co-Author, Co-Author

*** Correspondence:** Corresponding Author: email@uni.edu

# Supplementary Figures and Tables

## Supplementary Figures


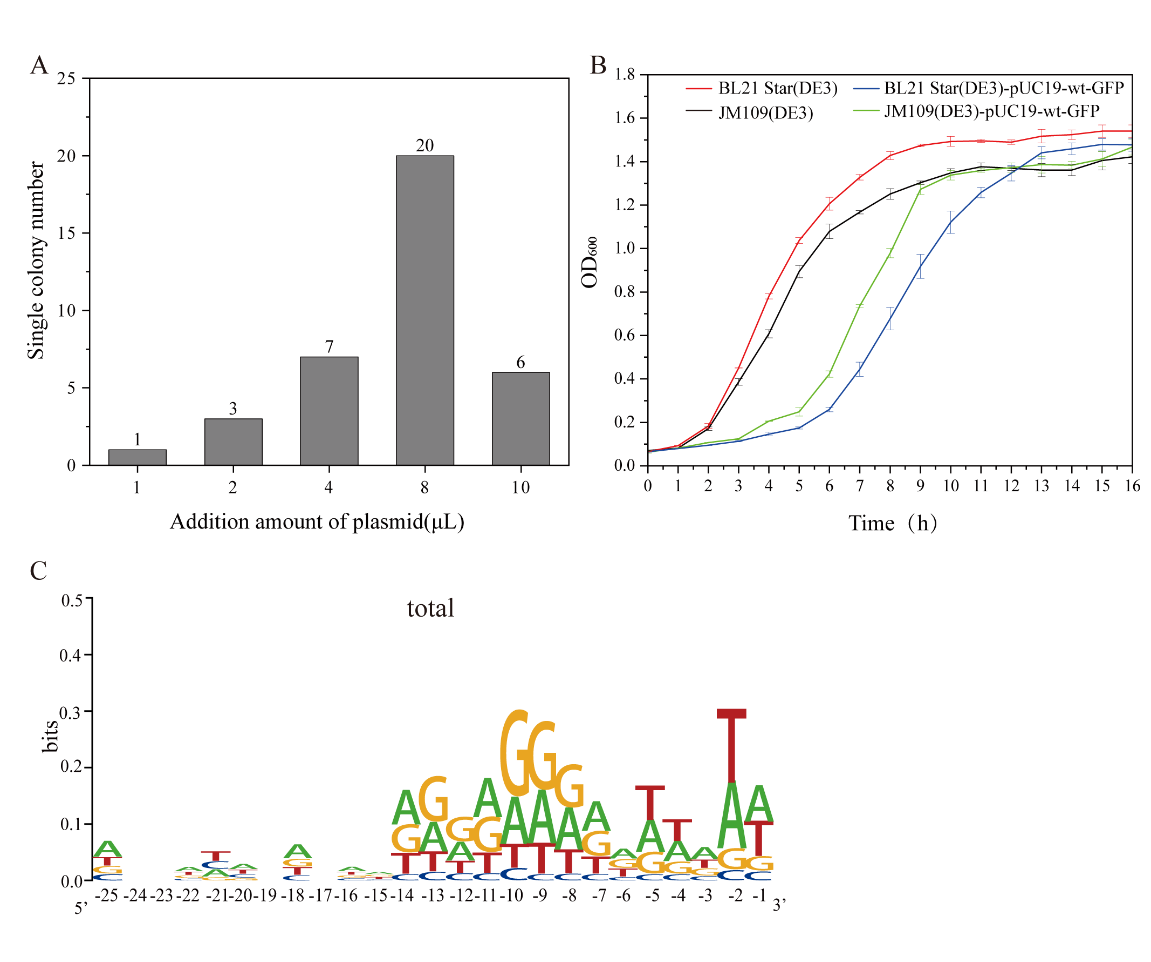


**Supplementary Figure 1.** The characterization and analysis for the 5'-UTRs measurement in vivo. (A) The optimized volume for the obtained plasmid DNA during the transformation. (B) The respective growth curve of themselves and the transformed benchmark plasmid for both the *E.coli* JM109(DE3) and *E.coli* BL21 Star (DE3) strains. (C) The sequence logo calculated for total of 160 unique 5'-UTR sequences.


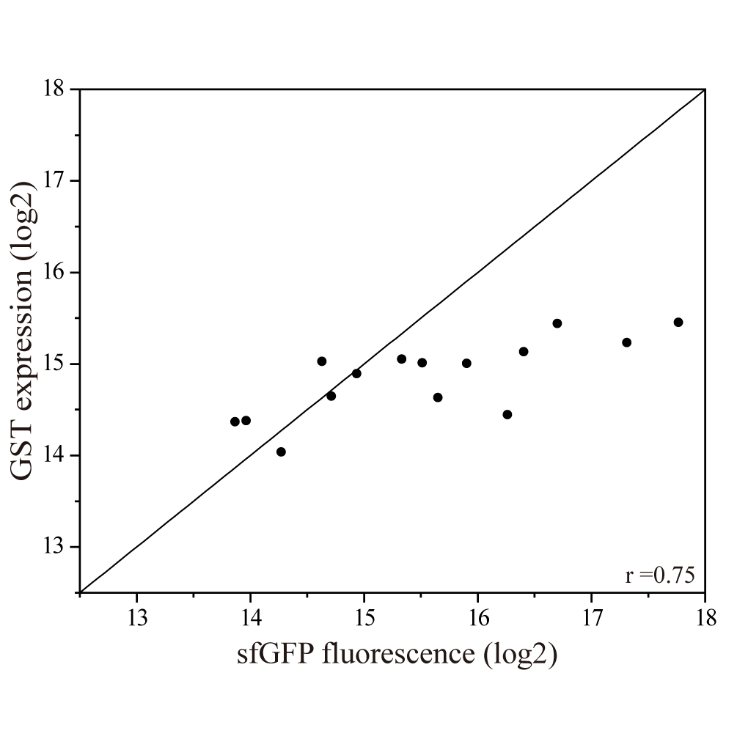


**Supplementary Figure 2.** The characterization for the 15 different 5'-UTR sequences between the GST reporter and sfGFP reporter in BL21 Star (DE3). we successfully constructed 15 different 5'-UTR variants with the reporter protein GST in BL21 Star (DE3) cell. To facilitate the determination of GST protein expression, Strep-Tag II was added to the C-terminal of GST protein. Under the OD_600_ of the bacterial solution was 0.6, the bacteria were collected and then the western blotting method to quantify the gray value of the target bands for determining the expression level of GST protein. Surprisingly, the GST reporter and sfGFP reporter for the 15 different 5'-UTR sequences in E. coli B strain showed a medium correlation (Pearson’s r = 0.75).


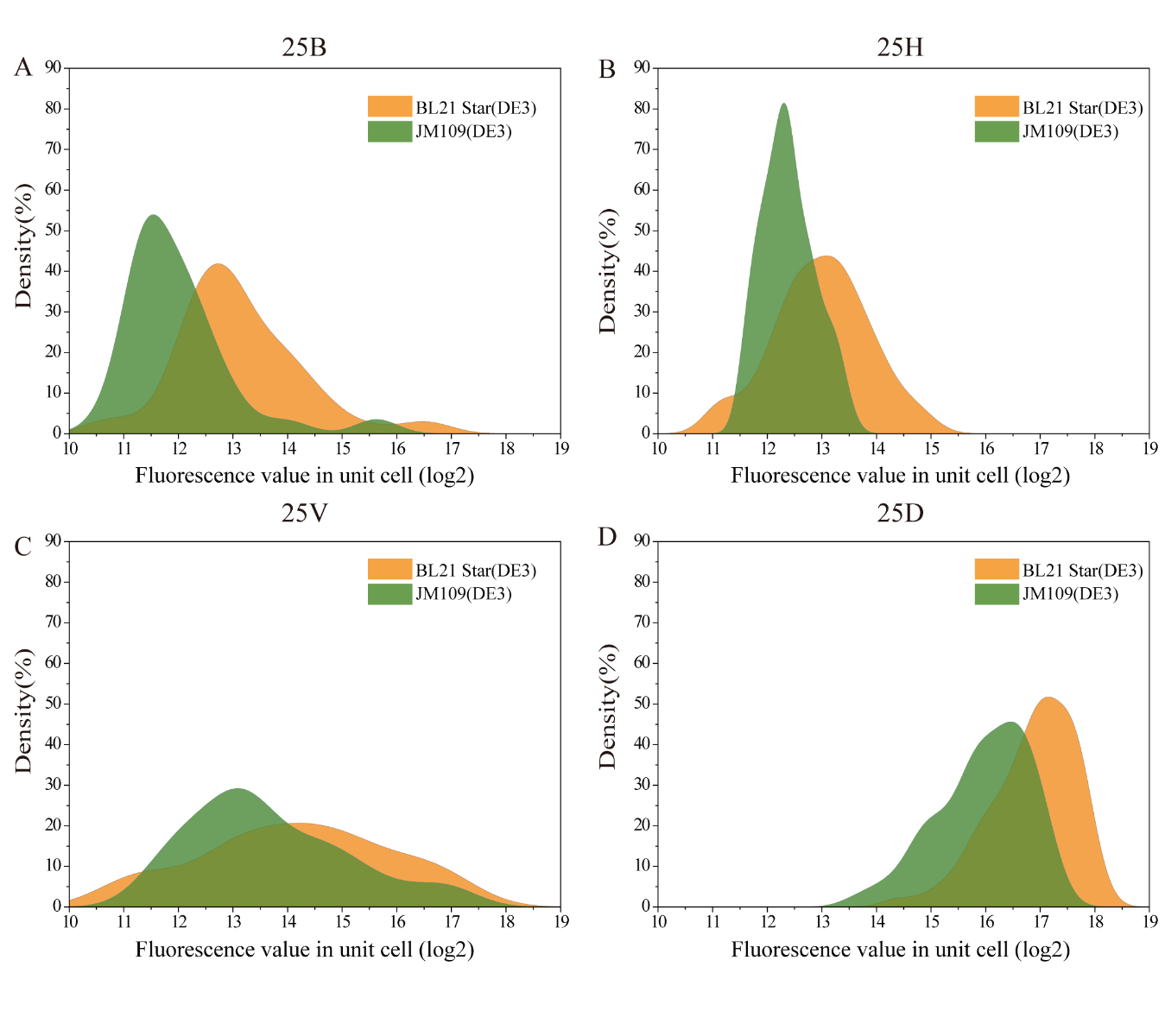


**Supplementary Figure 3.** The distribution of absolute fluorescence for the 5'-UTRs biased library between the *E.coli* JM109(DE3) cell and *E.coli* BL21 Star (DE3) cell. (A) The 25B library consist of 59 unique 5'-UTRs sequence. (B) The 25H library consist of 63 unique 5'-UTRs sequence. (C) The 25V library consist of 68 unique 5'-UTRs sequence. (D) The 25D library consist of 66 unique 5'-UTRs sequence.


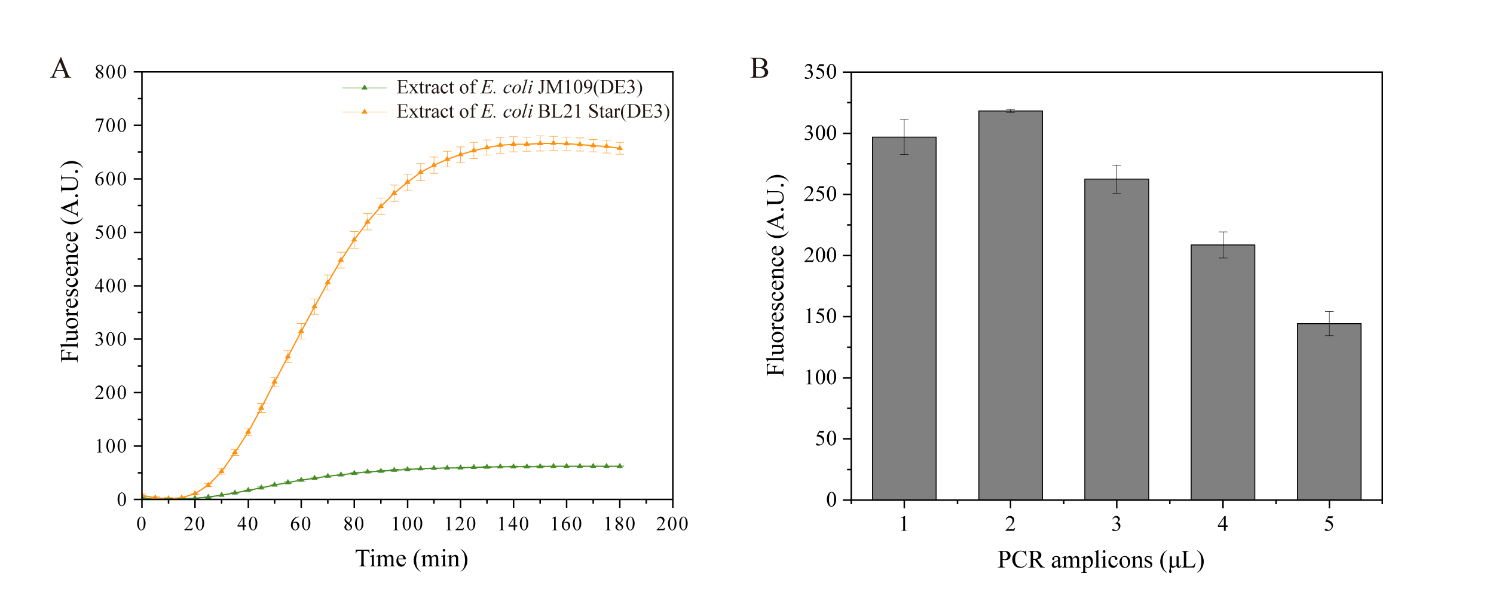


**Supplementary Figure 4.** Evaluation and optimization of sfGFP synthesis level in vitro expression system. (A) The activity determination of the sfGFP expression level in vitro using the 600ng purified PCR products with the benchmark 5'-UTRs sequence. (B) The optimized volume for the directly additive PCR amplicons in CFPS system.


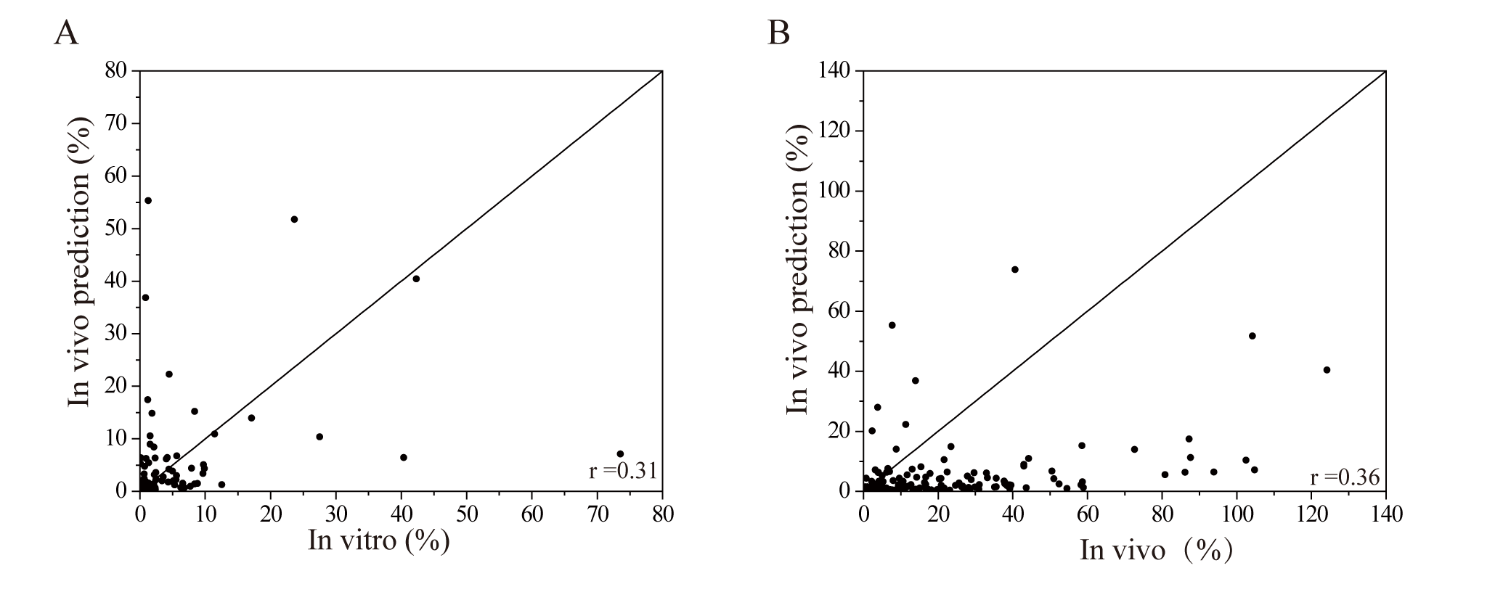


**Supplementary Figure 5.** The correlation analysis for 93 unique 5'-UTRs sequence predictions based on the different platform of the *E.coli* BL21 Star (DE3) strain. (A) The correlation analysis of 5'-UTRs of BL21 Star (DE3) in vivo prediction and in vitro system (Pearson’s r = 0.31). (B) The correlation analysis of 5'-UTRs of BL21 Star (DE3) in vivo prediction and in living cell system (Pearson’s r = 0.36). Pearson’s r shown at bottom right.

## Supplementary Tables

Table S1. List of primers in this work.

| Name | Sequence (5' to 3') |
| --- | --- |
| 25N-F | GGCAGTGAGCGCAACGCAATGCGCTAATACGACTCACTATAGGGNNNNNNNNNNNNNNNNNNNNNNNNNATGCGTAAAGGCGAAGAGCT |
| 25B-F | GGCAGTGAGCGCAACGCAATGCGCTAATACGACTCACTATAGGGBBBBBBBBBBBBBBBBBBBBBBBBBATGCGTAAAGGCGAAGAGCT |
| 25H-F | GGCAGTGAGCGCAACGCAATGCGCTAATACGACTCACTATAGGGHHHHHHHHHHHHHHHHHHHHHHHHHATGCGTAAAGGCGAAGAGCT |
| 25V-F | GGCAGTGAGCGCAACGCAATGCGCTAATACGACTCACTATAGGGVVVVVVVVVVVVVVVVVVVVVVVVVATGCGTAAAGGCGAAGAGCT |
| 25D-F | GGCAGTGAGCGCAACGCAATGCGCTAATACGACTCACTATAGGGDDDDDDDDDDDDDDDDDDDDDDDDDATGCGTAAAGGCGAAGAGCT |
| 25-R | AGCTCTTCGCCTTTACGCAT |
| V19-F | ATGCGTAAAGGCGAAGAGCT |
| V19-R | ATTGCGTTGCGCTCACTGCCC |
| F2 | TCCCGACTGGAAAGCGGGCAGTGAGCGCAACGCAATGCGCTAATACGACTCACTATAGGG |
| R2 | GTTCCTCCTTTCAGCAAAAAACCCCTCAAGACCCGTTTAGAGGCCCCAAGGGGTTATGCTAGTTATTGCTCAGCGGTGGCAGCAG |
